# White matter alterations in heart-kidney imbalance insomnia and Jiao-Tai-Wan treatment: A diffusion-tensor imaging study

**DOI:** 10.1007/s11682-022-00653-6

**Published:** 2022-03-25

**Authors:** Jie Chen, Yanxuan Li, Nengzhi Xia, Caiyun Wen, Tianyi Xia, Yuandi Zhuang, Mengmeng Jiang, Yilan Xiang, Mingyue Zhang, Chenyi Zhan, Yunjun Yang, Zhengzhong Yuan, Qun Huang

**Affiliations:** 1 Department of Radiology, Wenzhou Central Hospital, Wenzhou, 325000 China; 2 Department of Radiology, The First Affiliated Hospital of Wenzhou Medical University, Wenzhou, 325000 China; 3 Department of Traditional Chinese Medicine, The First Affiliated Hospital of Wenzhou Medical University, Wenzhou, 325000 China

**Keywords:** Insomnia, Jiao-tai-wan, Diffusion-tensor imaging, Magnetic resonance imaging, White matter

## Abstract

Previous studies have reported changes in white matter microstructures in patients with insomnia. However, few neuroimaging studies have focused specifically on white matter tracts in insomnia patients after having received treatment. In this prospective study, diffusion-tensor imaging was used in two samples of heart-kidney imbalance insomnia patients who were treated with placebo or Jiao-Tai-Wan, a traditional Chinese medicine commonly used to treat heart-kidney imbalance insomnia, to assess the changes in white matter tracts. Tract-based spatial statistical analyses were first applied to compare the changes in mean diffusivity and fractional anisotropy of white matter between 75 heart-kidney imbalance insomnia patients and 41 healthy control participants. In subsequent randomized, double-blind, placebo-controlled trials, comparisons of mean diffusivity and fractional anisotropy were also performed in 24 heart-kidney imbalance insomnia patients (8 males; 16 females; 42.5 ± 10.4 years) with Jiao-Tai-Wan and 26 heart-kidney imbalance insomnia patients (11 males; 15 females; 39.7 ± 9.4 years) with a placebo, with age and sex as covariates. Fractional anisotropy values in left corticospinal tract were increased in heart-kidney imbalance insomnia patients. Heart-kidney imbalance insomnia patients showed lower mean diffusivity and fractional anisotropy values of several white matter tracts than healthy control participants, such as the bilateral anterior limb of internal capsule, bilateral superior longitudinal fasciculus and bilateral posterior corona radiata. After being treated with Jiao-Tai-Wan, heart-kidney imbalance insomnia patients showed a trend towards reduced fractional anisotropy values in the left corticospinal tract. Jiao-Tai-Wan may improve the sleep quality by reversing the structural changes of the left corticospinal tract caused by heart-kidney imbalance insomnia.

## Introduction

Insomnia is one of the most prevalent subjective complaints of sleep-disordered patients worldwide, and it is reported that approximately 4–20% of the population suffers from insomnia (Morin et al. 2011; D. Riemann et al. 2011; Roth et al. 2011). The main characteristic of insomnia is that people with insomnia usually have chronic difficulty falling asleep, difficulty staying sleep, and/or early morning awakening. Insomnia is associated with an increased risk of psychiatric disorders and the quality of an affected patient’s life is severely reduced (Baglioni et al. 2011; Kyle et al. 2010; Morin et al. 2015). Moreover, chronic insomnia is a predisposing factor for type II diabetes, metabolic syndrome and cardio-cerebrovascular diseases (Knutson et al. 2007; M. Li et al. 2014). Although numerous studies have focused on insomnia, the mechanisms underlying the occurrence of insomnia remain poorly understood. Insomnia is often accompanied by other somatic symptoms. These signs and symptoms are diverse and reflect the malfunctioning or imbalance of various body systems, which could be either causes or consequences of insomnia. According to the signs and symptoms of patients, traditional Chinese medicine divides insomnia into many different patterns. The heart-kidney imbalance is one of the main patterns, often accompanied by red tongue, thin coating, fine and rapid pulse, dizziness, tinnitus, seminal emission, night sweating and so on. (Poon et al. 2012).

Current guidelines set cognitive behavioral therapy for insomnia as the preferred treatment for patients who suffer from insomnia (Buysse. 2013; Schutte-Rodin et al. 2008). However, poor adherence may diminish its therapeutic effect (Matthews et al. 2013). Many people cannot address their insomnia without taking a combination of medications. Besides, most therapies only transiently alleviate the symptoms of insomnia instead of delivering a complete cure. The most widely used medications for insomnia are benzodiazepines and benzodiazepine receptor agonists (Buysse. 2013). Still, these therapies incur numerous side effects, including depression, poor memory or forgetfulness, drowsiness and impaired work productivity. In addition, their use is heavily limited by developed tolerance and increased dependency with long-term treatment (Buysse. 2013; Dieter Riemann et al. 2015). So far, guidance for clinicians in choosing the best treatment remains limited, and the search for better treatments is underway. In recent years, increasing evidence has shown that traditional Chinese medicine can be effectively applied in the treatment of insomnia (Singh and Zhao, 2017; Yeung et al. 2012; Zhang et al. 2019). It was reported that Jiao-tai-wan (JTW), made of Rhizome Coptidis and Cortex Cinnamomi at a ratio of 10:1, has a remarkable effect in treating of insomnia linked to an incompatibility between heart and kidney (Huang et al. 2020; Sun et al. 2020; Yue et al. 2016). Experimental studies have shown that JTW can not only regulate levels of neurotransmitters in the blood, such as orexin A (brain tissue), 5-hydroxy tryptamine and γ-aminobutyric acid, which are all therapeutic targets for insomnia (Abad and Guilleminault, 2018; Huang et al. 2020), but it may also regulate immune cytokines, such as interleukin-6 and tumor necrosis factor, which are upregulated by sleep loss (Zou et al. 2017).

Diffusion-tensor imaging (DTI) is a non-invasive method which is widely used to assess alterations in white matter integrity. The decrease of fractional anisotropy (FA) value means the destruction of white matter integrity. Although several studies have investigated white matter structures in people with insomnia, the results of these studies remained inconsistent. For instance, Spiegelhalder et al. (Spiegelhalder et al. 2014) reported a reduced white matter integrity of bilateral anterior internal capsule in primary insomnia, while Bresser et al. (Bresser et al. 2020) only found decreased FA values in the right limb of the anterior internal capsule. Kang et al. (Kang et al. 2018) showed low white-matter integrity between the inferior frontal gyrus and left thalamus in insomnia patients. Li et al. (S. Li et al. 2016) indicated that the integrity of right lateralized white matter in insomnia patients was reduced. Sexton et al. (Sexton et al. 2017) even reported that poor sleep quality was correlated with reduced global FA values. Besides, the effect of insomnia treatment on white matter has not yet been studied.

Therefore, we sought to investigate the alterations in white-matter structure caused by insomnia, and the therapeutic effect of JTW in insomnia based on evidence of white matter integrity.

## Methods

### Participants

This prospective study was designed as a double-blind, randomized, placebo-controlled trial and was approved by the Institutional Medical Ethics Committee of No. 1 Affiliated Hospital, Wenzhou Medical University (Approval document #2016045). Heart-kidney imbalance insomnia patients (HKIIPs) were recruited from The First Affiliated Hospital of Wenzhou Medical University from September 2018 to February 2020 through WeChat, flyers and bulletin boards at the hospital. All HKIIPs were asked to complete the magnetic resonance imaging (MRI) examination, polysomnography (PSG) and Pittsburgh Sleep Quality Index (PSQI) before and after the medication intervention.

The inclusion criteria for HKIIPs were as follows: (a) Aged 18- to 60- years-old, right-handed, male and female, in junior high school or above; (b) According to the DSM-V, insomnia was defined as having difficulty falling asleep, staying asleep or having refreshing sleep, occurring 3 or more times per week, for at least 3 months (Seow et al. 2018); (c) A PSQI score of greater than or equal to 7 according to the criteria for insomnia described in CCMD-3 (Zeng et al. 2020); (d) A Disharmony of Heart and Kidney Scoring System score is greater than 9 according to the “Guide Principles for Clinical Research of New Drugs of TCM” (Zeng et al. 2020); (e) Free of any psychoactive medication or hypnotic or cognitive behavioral therapy for insomnia for at least 2 weeks before the study.

Major exclusion criteria are listed as follows: (a) The presence of any abnormal brain MRI findings; (b) Insomnia caused by changes in lifestyle or environmental factors, such as moving, night shift, noise, vigorous exercise before going to bed; (c) A history of serious mental disorders (e.g., anxiety, depression or schizophrenia); (d) Suffering from a physical disorder that affects the central nervous system; (e) Having liver and kidney dysfunction; (f) Alcoholism and/or psychotropic drug addiction; (g) Pregnant, breastfeeding or menstruating women.

**Fig. 1:**
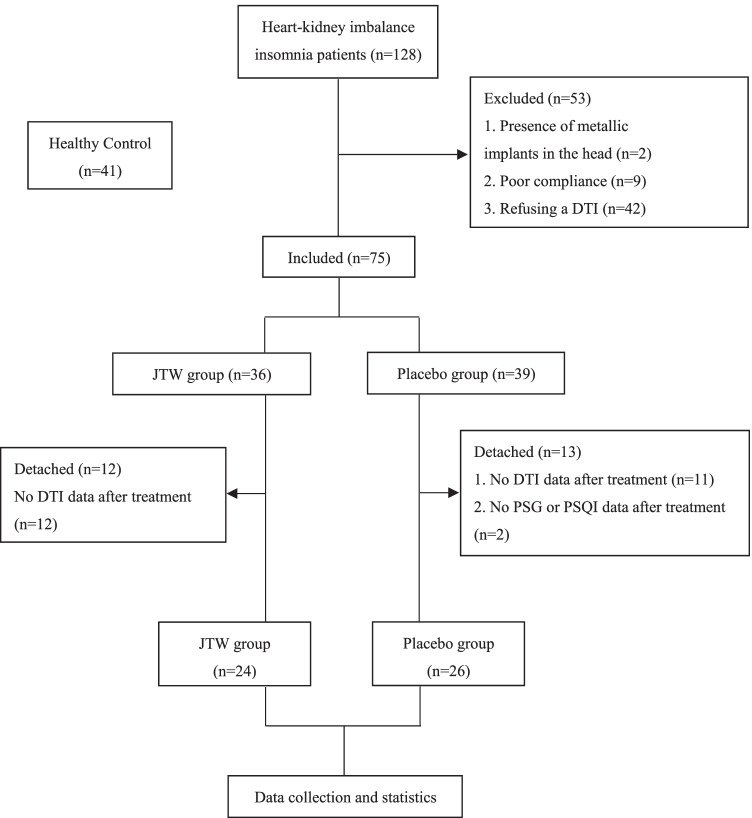
Flowchart representing study patients’ selection.

The brief flow diagram of the study process is shown in Fig. 1. A total of 128 HKIIPs who passed preliminary screening criteria were recruited to this study. Among these, forty-two patients were excluded because of refusing DTI, two due to the presence of metallic implants in the head and nine due to poor compliance. Seventy-five eligible HKIIPs (26 males, 49 females) were included in the present study. HKIIPs were randomly divided into two groups: the JTW group (n = 36) and the placebo group (n = 39). A total of 25 patients were excluded based on the following criteria: no DTI data after treatment (JTW group =12; placebo group= 11); lack of post-treatment PSQI and PSG data (JTW group =0; placebo group= 2). Eventually, we obtained a sample of 50 HKIIPs (JTW group =24, placebo group= 26; 19 men, 31 women).

Forty-one age- and sex-matched healthy controls (HCs; 16 males, 25 females) were also recruited from the local community through advertisements as mentioned above. The inclusion criteria for HC participants were as follows : good sleep quality (PSQI≤5); no history of psychiatric or neurologic diseases; and normal conventional MRI findings.

All participants for examination and/or treatment were asked to sign an informed consent form.

### Intervention

A double-blind placebo-controlled drug administration technique was used. Both experimental drugs (JTW) and matched placebos were made by the Kang-ren Pharmaceutical Factory (Shanghai China). JTW were produced as yellowish-brown granules, containing 1.1 g JTW soft extract consisted of Cortex Cinnamomi and Rhizome Coptidis at a ratio of 1:10. The placebos (containing only the excipients, i.e., corn starch, citric acid, lactose hydrate and caramel color) with the same appearance and color as JTW were packaged in identical plastic bags to ensure blinding. HKIIPs ingested 2 g JTW or placebo at 4pm and 9pm daily for a week according to groups, and followed the scheduled examination time at the termination of the medication intervention period.

### Disharmony of Heart and Kidney Scoring System score

The Disharmony of Heart and Kidney Scoring System score include upset, palpitation, dizziness, tinnitus, sour waist, soft legs, hand and foot heart heat, hot flashes, night sweats, dry mouth and pharynx, seminal emission / irregular menstruation, red tongue, thin coating, fine and rapid pulse. According to the “Guide Principles for Clinical Research of New Drugs of TCM”, the clinical symptoms mentioned above are divided into four grades according to severity: none, mild, moderate and severe, and are rated as 0, 1, 2 and 3 respectively. The higher the score, the more serious the symptoms are.

### Pittsburgh Sleep Quality Index

The Pittsburgh Sleep Quality Index (PSQI) used in this study is a retrospective self-rated questionnaire consisting of 19 items (Buysse et al. 1989). A higher PSQI score means worse quality of sleep. PSQI was firstly used to compare the sleep quality of HKIIPs and HCs. PSQI was also performed before and one month after the start of medicine intervention to evaluate the curative effect of JTW.

### Polysomnography

Polysomnography (PSG) was performed at baseline and a week later at the end of the medication intervention. All HKIIPs underwent full, in-laboratory PSG by study-certified technicians according to the guidelines of American Academy of Sleep Medicine. Changes in sleep quality were objectively measured by mean changes in all parameters, including total sleep time (TST), sleep efficiency (SE), sleep onset latency (SL), rapid eye movement latency (RL), wake-time after sleep onset (WASO), awakening times (AT), arousal index (AI), rapid eye movement (REM) and 3 parts of non-rapid eye movement (N1, N2 and N3), from baseline to one week later at the end of the medication intervention. During the recording period, all participants were asked to abstain from alcohol, caffeine and daytime naps.

### MRI

Each participant signed a written consent form before undergoing the MRI examination. All MRI scans were carried out using a 3 Tesla MRI scanner (Philips Achieva TX) with an 8-channel receive-only head coil. Additionally, in order to reduce the effect of scanner noise and head motion, all subjects laid in a supine position with ear plugs and foam pads. The DTI datasets were acquired along 32 gradient directions (b = 1000 s/mm^2^), including five acquisitions without diffusion weighting (b = 0). The sequence parameters were as follows: repetition time (msec) / echo time (msec), 6800 / 93; 128 × 128 matrix; field of view, 256 mm × 256 mm; slice thickness = 3 mm, no gap; and 50 contiguous axial slices. Several other sequences were also scanned, such as T2-weighted images, T1-weighted images, T1- fluid attenuated inversion recovery (FLAIR) images, T2-FLAIR images, and all scans were inspected for motion artefacts and for the absence of pathologic findings by a neuroradiologist.

### Data analysis

Data preprocessing —DTI data were preprocessed using tools in FSL (FMRIB Software Library 5.0.9) as follows. Firstly, all DTI images were corrected for distortions caused by eddy current distortions and head motion by using an affine alignment of each diffusion-weighted image to the non-diffusion weighted (b = 0) image by the FMRIB’s Diffusion Toolbox. Secondly, FSL's Brain Extraction Tool (BET) was applied to generate a binary mask from the b0 image and remove any non-brain tissue (fractional intensity threshold = 0.2) (Stephen M. Smith. 2002). Finally, a tensor model was fitted locally to each voxel using DTIfit (FSL) and maps of FA and MD were calculated.

Tract-based spatial statistics —The voxel-wise statistical analysis of DTI data was based on tract-based spatial statistics (TBSS) from the FMRIB Software Library (S. M. Smith et al. 2006). The FA images from all subjects (n = 116) were aligned to a common target FA image (1 × 1 × 1 mm^3^ MNI152 FMRIB58_FA standard space) using a non-linear registration. Next, a mean FA image was created and a mean FA skeleton map thresholded to the standard value of 0.2 was generated, representing the centers of all tracts. Subsequently, each participant’s aligned FA images were projected onto the mean FA skeleton for statistical analysis. For better assessment, a TBSS analysis was repeated for MD maps.

### Statistical analysis

Differences in age, PSQI and objective sleep measures on polysomnography between HKIIPs and HCs, JTW and placebo group were analyzed by two-sample, two-tailed t tests. Differences in the proportion of females and males between above groups were determined by a two- tailed Pearson χ2 test. Differences in clinical efficacy of medication between JTW and placebo group were assessed by ANCOVA.

A white-matter statistical analysis (covarying for age and sex) was firstly conducted using a general linear model in the FSL randomize tool. Then, 5000 permutation analysis of linear models were carried out with MATLAB software. Finally, threshold-free cluster enhancement (TFCE) was applied to define significant clusters (*p* < 0.05) and correct multiple comparisons for family-wise error (FWE). Cluster-correction (CC, *p* < 0.01 and a minimum cluster size of 50 voxels) was used, when FWE correction showed no significant clusters. The Johns Hopkins University (JHU) ICBM-DTI81 White Mater Labels and JHU white-matter tractography atlas were used to locate the specific fibers.

## Results

### Demographic characteristics

Table 1 presents the characteristics of the participants in this study. The differences between HC group and HKIIPs group were not significantly different for age (*p* = 0.641) or sex distribution (*p* = 0.816). Furthermore, HKIIPs had higher PSQI scores than HC participants (12.5±3.0 versus. 2.9±1.4; *p < 0.001*).

**Table 1:** Demographics and clinical characteristics of all Participants

	All participants					HKIIPs with treatment	
	HKIIPs	HC	*P*		Placebo group	JTW group	*p*
Sex (M/F)	26/49	16/25	0.641		11/15	8/16	0.514
Age (years)	40.3±9.9	40.9±16.1	0.816		39.7±9.4	42.5±10.4	0.328
PQSI	12.5±3.0	2.9±1.4	<0.001^**^		11.7±2.9	12.5±3.2	0.401
PSG							
TST (min)	-	-	-		360.1±110.1	374.9±96.0	0.616
SE (%)	-	-	-		67.6±20.8	69.4±16.7	0.740
SL (min)	-	-	-		65.0±72.1	46.5±44.6	0.286
RL (min)	-	-	-		120.3±71.3	170.1±102.1	0.050
WASO (min)	-	-	-	-	109.2±92.0	118.4±72.6	0.699
AT	-	-	-		15.9±18.4	9.2±8.7	0.107
AI (/hr)	-	-	-		9.7±5.5	9.3±3.8	0.764
N 1 (%)	-	-	-		6.9±3.9	8.1±3.9	0.273
N 2 (%)	-	-	-		61.7±10.3	60.8±14.1	0.796
N 3 (%)	-	-	-		16.3±8.9	17.2±13.4	0.776
REM (%)	-	-	-		15.1±6.3	13.9±4.4	0.425

^**^p<0.001; HKIIPs = heart-kidney imbalance insomnia patients; HC = Healthy control; JTW = Jiao-Tai-Wan; PQSI = Pittsburgh Sleep Quality Index; PSG = polysomnography; TST = Total sleep time; SE = Sleep efficiency; SL = Sleep onset latency; RL = Rapid eye movement latency; WASO =Wake-time after sleep onset; AT = Awakening times; AI = Arousal index; N =Non-rapid eye movement; REM = Rapid eye movement.

### Whole brain white matter difference

#### Comparison between HCs and HKIIPs

**Fig. 2:**
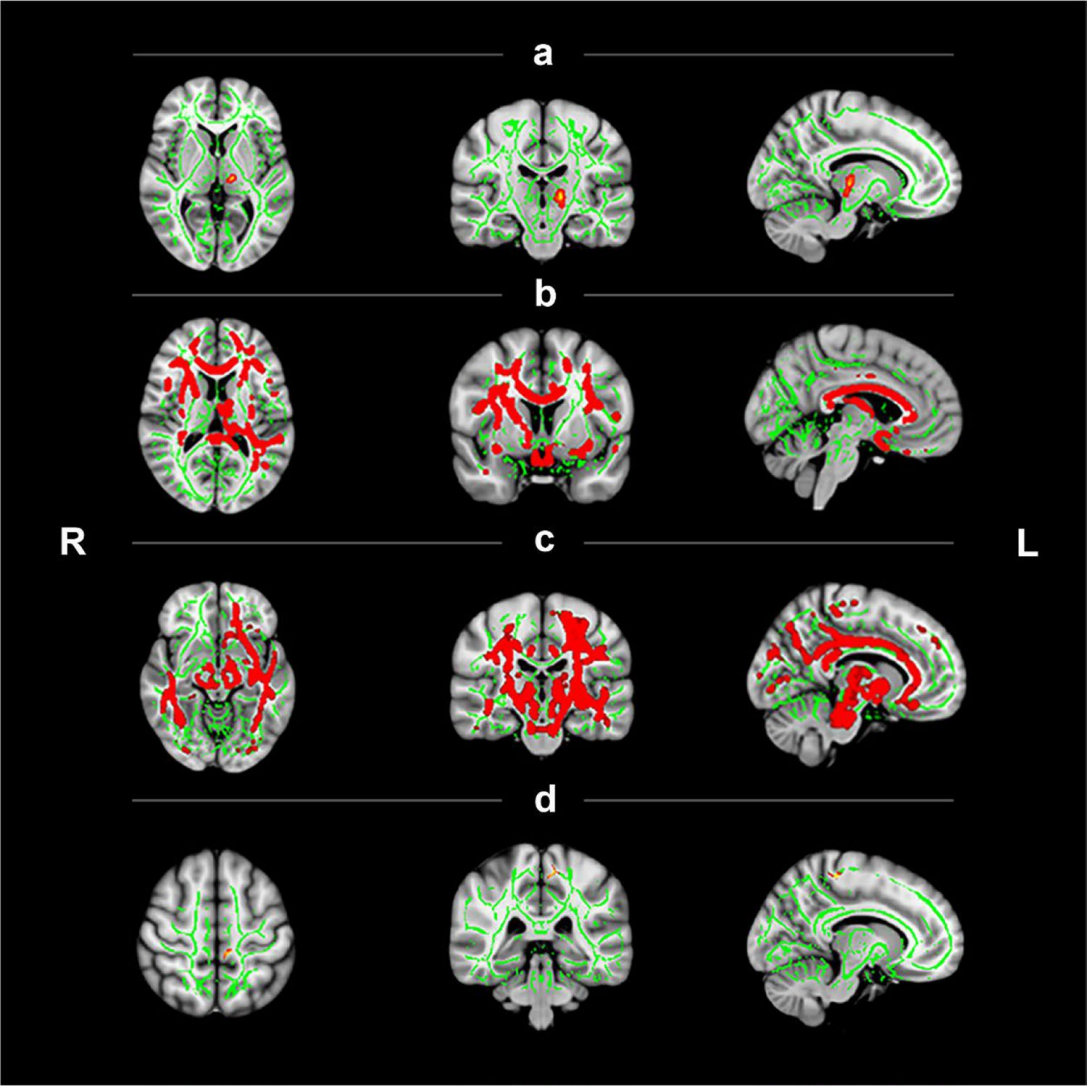
Tract-based spatial statistics analysis showing increased or decreased fractional anisotropy (FA) and mean diffusivity (MD) values in different white matter (WM) tracts of heart-kidney imbalance insomnia patients (HKIIPs). Green represents the mean FA or MD skeleton across all participants. Red yellow depicts the WM tracts whose FA or MD values were significantly changed (family-wise error correction, FWE, *p* < 0 .05 or cluster-correction, CC, *p* < 0.01 and cluster size > 50 voxels). (**A**) WM tracts with increased FA values in HKIIPs compared with health control (HC) (FWE, *p* < 0 .05); (**B**) WM tracts with decreased FA values in HKIIPs compared with HC (FWE, *p* < 0 .05); (**C**) WM tracts with decreased MD values in HKIIPs compared with HC (FWE, *p* < 0 .05); (**D**) WM tracts with decreased FA values in JTW group compared with placebo group (CC, *p* < 0.01 and cluster size > 50 voxels).

As shown in Fig. 2 and Table 2, the tract-based spatial statistics analysis demonstrated that when compared with HCs, HKIIPs showed significantly higher FA values in the left corticospinal tract (CST) (Fig. 2A, FWE, *p* < 0.05 ), and significantly lower FA values in several other white matter tracts, including the bilateral superior longitudinal fasciculus (SLF), bilateral anterior limb of internal capsule (ALIC), bilateral anterior and posterior corona radiata (ACR and PCR), bilateral body of corpus callosum (BCC), left cingulum, left posterior thalamic radiation (PTR), right superior corona radiata (SCR), and right CST (Fig. 2B, FWE, *p* < 0.05). In addition, the tract-based spatial statistics analysis showed lower MD values in HKIIPs at several fibers, such as bilateral SLF, bilateral PCR, bilateral cingulum, bilateral cerebral peduncle, bilateral CST, bilateral ALIC, bilateral posterior limb of internal capsule, bilateral PTR, bilateral SCR, left ACR, and left BCC (Fig. 2C, FWE, *p* < 0.05).

**Table 2:** Results of white matter analysis

Contrast	L/R	Peak region	Cluster size	MNI coordinate		
				X	Y	Z
HKIIPs -HC (FA)	L	CST	47	105	105	69
HC- HKIIPs (FA)			33259			
	L/R	SLF		127/53	95/95	104/105
	L/R	ALIC		108/71	141/142	79/79
	L/R	ACR		113/64	151/150	79/79
	L/R	PCR		116/64	95/95	100/100
	L/R	BCC		102/74	120/118	102/108
	L	Cingulum		97	112	107
	L	PTR		126	70	74
	R	SCR		72	136	106
	R	CST		80	107	50
HC- HKIIPs (MD)			49525			
	L/R	SLF		126/54	100/100	101/100
	L/R	PCR		116/64	99/100	101/101
	L/R	Cingulum		98/82	111/110	106/106
	L/R	CP		102/77	102/102	62/62
	L/R	CST		98/82	101/101	44/44
	L/R	ALIC		107/73	126/126	82/82
	L/R	PLIC		114/66	108/107	82/82
	L/R	PTR		118/61	58/57	84/84
	L/R	SCR		108/73	137/137	109/109
	L	ACR		104	158	62
	L	BCC		106	119	106
BJT-AJT (FA)	L	CST	85	95	92	127

HKIIPs = heart-kidney imbalance insomnia patients; HC = Healthy control; FA = fractional anisotropy; MD = mean diffusivity; BJT = Before JTW Treatment; AJT = After JTW Treatment; CST = corticospinal tract; SLF = Superior longitudinal fasciculus; ALIC = Anterior limb of internal capsule; ACR = Anterior corona radiata; PCR = Posterior corona radiata; BCC = Body of corpus callosum; PTR = Posterior thalamic radiation; SCR = Superior corona radiata; CP = Cerebral peduncle; PLIC = Posterior limb of internal capsule.

#### Clinical efficacy after medication

The characteristics of the JTW group and the placebo group are shown in Table 1. There were no significant differences in gender, age, PSQI score or PSG between the two groups at baseline. PSQI and PSG regulated by ANCOVA were showed in Table 3. PSQI score of HKIIPs decreased significantly after treatment with JTW, but no changes were observed for PSG. Additionally, in Fig. 2D, HKIIPs showed decreased FA values in the left CST after JTW treatment, while the white matter structure of the placebo group remained unchanged (CC, *p* < 0.01 and cluster size > 50 voxels).

**Table 3:** Differences in clinical efficacy of medication

	Placebo group	JTW group	*P*
PSQI			0.003*
Baseline	11.7±2.9	12.5±3.2	
Post-treatment^a^	10.9±0.5	8.7±0.5	
PSG			0.500
TST (min)			
Baseline	360.1±110.1	374.9±96.0	
Post-treatment^a^	421.6±14.6	407.2±15.2	
SE (%)			0.584
Baseline	67.6±20.8	69.4±16.7	
Post-treatment^a^	77.8±2.5	75.8±2.6	
SL (min)			0.946
Baseline	65.0±72.1	46.5±44.6	
Post-treatment^a^	57.7±12.6	58.9±13.1	
RL (min)			0.836
Baseline	120.3±71.3	170.1±102.1	
Post-treatment^a^	126.0±12.9	122.1±13.5	
WASO (min)			0.274
Baseline	109.2±92.0	118.4±72.6	
Post-treatment^a^	57.6±10.4	74.2±10.8	
AT			0.195
Baseline	15.9±18.4	9.2±8.7	
Post-treatment^a^	18.8±2.7	13.5±2.8	
AI (/hr)			0.965
Baseline	9.7±5.5	9.3±3.8	
Post-treatment^a^	9.7±0.7	9.8±0.8	
N 1 (%)			0.078
Baseline	6.9±3.9	8.1±3.9	
Post-treatment^a^	7.5±0.8	5.5±0.8	
N 2 (%)			0.195
Baseline	61.7±10.3	60.8±14.1	
Post-treatment^a^	58.2±2.4	62.6±2.4	
N 3 (%)			0.248
Baseline	16.3±8.9	17.2±13.4	
Post-treatment^a^	18.9±2.2	15.2±2.3	
REM (%)			0.708
Baseline	15.1±6.3	13.9±4.4	
Post-treatment^a^	16.1±0.9	16.6±1.0	

^*^p<0.01; ^a^ regulated by ANCOVA; JTW = Jiao-Tai-Wan; PQSI = Pittsburgh Sleep Quality Index; PSG = polysomnography; TST = Total sleep time; SE = Sleep efficiency; SL = Sleep onset latency; RL = Rapid eye movement latency; WASO =Wake-time after sleep onset; AT = awakening times; AI = Arousal index; N = Non-rapid eye movement; REM = Rapid eye movement.

## Discussion

Previous studies mainly focused on the changes of white matter in patients with primary insomnia. In this study, we assessed the differences in white matter integrity between HKIIPs and HCs, in addition to the curative effect of JTW on white matter integrity in HKIIPs.

### Analysis of white matter changes in HKIIPs

Our findings showed increased FA values in the left CST of HKIIPs compared with HCs. We speculate that this may be associated with increased cortical excitability. The hyperarousal theory of insomnia assumes that increased cognitive and physiological arousal leads to difficulty in initiating or maintaining sleep (Perlis et al. 1997). A study of cortical excitability indicated that chronic insomnia patients showed a globally increased excitability, with larger motor evoked potential sizes to stimulation compared with control participants (van der Werf et al. 2010). In addition, it is reported that a lower corticospinal excitability is related to a smaller CST volume (Lepley et al. 2020). Considering that the CST is of paramount importance in the somatic motor system and increased FA values can be attributable to improved axon density and fiber coherence, we boldly speculated that the increased FA of CST in patients with insomnia was associated with increased cortical excitability. Autonomic, neuroendocrine, electrophysiological, neuroimmunological and neuroimaging findings confirm increased arousal levels in patients with primary insomnia (Bonnet and Arand, 2010; D. Riemann et al. 2010). Insomniacs showed nocturnal elevations of norepinephrine (M. Irwin et al. 2003). Norepinephrine not only has neuroprotective and anti-inflammatory effects, but also can promote the differentiation of oligodendrocytes, and then promote the formation of myelin (Galea et al. 2003; Ghiani et al. 1999; Madrigal et al. 2007). With the formation of myelin, the FA value of neural fibers may increase (Beaulieu. 2002). Conversely, our study also found that the FA and MD values of a large number of fiber bundles in bilateral brain were decreased in HKIIPs. One possible explanation for our findings is the presence of insomnia-associated neuroinflammation involving white matter. A meta-analysis showed that insomnia is associated with increased systemic inflammation markers (M. R. Irwin et al. 2016). In addition, the proinflammatory interleukin IL-6 was highly expressed in the brain of sleep-disordered mice (Zhu et al. 2012). Higher levels of circulating inflammatory markers are correlated with lower FA values in the brain (Rodrigue et al. 2019). Neuroinflammation and low FA values have also been reported in mouse models of craniocerebral trauma (Missault et al. 2019). Thus, we believe that the decrease in FA and MD in the white matter of HKIIPs may be due to neuroinflammation. It is worth noting that the FA values of bilateral CST in HKIIPs showed inconsistent trends, whereby the FA value of right CST was decreased, while the left showed the opposite pattern. This may be due to the fact that the amount by which the FA value increased, due to the excitability in the dominant hemisphere, was much greater than the amount by which it decreased due to neuroinflammation.

### The therapeutic effect of JTW

We found that the FA value in the left CST was decreased in HKIIPs after JTW treatment. This may be attributed to the central inhibitory effect of JTW leading to decreased cortical excitability. JTW antagonizes central excitability by regulating the levels of various neurotransmitters and hormones associated with insomnia (Sun et al. 2020). It plays a sedative and hypnotic role by increasing gamma-aminobutyric acid content and receptor expression in the brain of rats. It also increases the level of 5-hydroxytryptamine and decreases the level of norepinephrine in the brain by inhibiting the hypothalamic–pituitary–adrenal axis (HPA) of insomnia rats, thus exerting its therapeutic effect on insomnia (Huang et al. 2020). Our findings may serve to elucidate the underlying therapeutic mechanism of JTW.

### PSQI and PSG results

The PSQI score of HKIIPs decreased significantly after treatment with JTW, reflecting the effectiveness of JTW in treating insomnia. The PSQI score was used to assess sleep quality and severity of sleep disorders in the past one month, while the PSG was mainly used to identify the sleep phases, such as REM, NREM, and wakefulness (Chen et al. 2018; Zeng et al. 2020). The PSG results showed no significant difference between the JTW and placebo groups after treatment, which could be due to the little effect of JTW on the sleep structure, or short-term treatment that has no effect on the structure of sleep. Better results might be got if giving a longer course of treatment.

### Limitations

This study has several limitations. Firstly, the sample size for the HKIIPs was too small. Secondly, the duration of treatment was not long enough, which may lead to no difference in PSG changes between JTW and placebo after treatment.

## Conclusion

Our study demonstrates that JTW treatment for one week did not affect objective sleep measures while the effects on subjective measures deserve further research. The FA value of multiple white matter in the whole brain of the HKIIPs decreased, while the FA value of the left CST increased. After one week of JTW treatment, the increased FA value of left CST decreased.

## Declarations

### Trial registration

Chinese Clinical Trial Registry, ChiCTR1800019239; registered on 1 November 2018-retrospectively registered, http://www.chictr.org.cn/listbycreater.aspx, more than a month after the start of the experiment. The delay was due to lack of experience regarding trail, registration with a data sharing website.
